# Cor triatriatum presenting as heart failure with reduced ejection fraction: a case report

**DOI:** 10.1186/1749-8090-6-83

**Published:** 2011-06-14

**Authors:** John Kokotsakis, Vania Anagnostakou, George Almpanis, Ioannis Paralikas, Ioannis Nenekidis, Theodoros Kratimenos, Efi Prapa, Nikolitsa Tragotsalou, Achilleas Lioulias, Andreas Mazarakis

**Affiliations:** 1 Cardiac Surgery Department, Evaggelismos General Hospital, Athens, Greece; 2 Radiology Department, Evaggelismos General Hospital, Athens, Greece; 3 1st Cardiology Department, Agios Andreas General Hospital, Patra, Greece

## Abstract

Cor triatriatum is a rare congenital cardiac malformation and it usually refers to the left atrium. We report an unusual case of cor triatriatum in a 33 - year old woman presented with congestive heart failure caused by left ventricular systolic dysfunction.

## Background

Cor triatriatum is a rare congenital cardiac malformation with an estimated incidence of 0,1% of all congenital heart disease and it usually refers to the left atrium (cor triatriatum sinister). In cor triatriatum sinister the left atrium is divided by a fibromuscular membrane into two distinct chambers: a posterior - superior chamber receiving the four pulmonary veins and an anterior - inferior chamber ( true left atrium ) that connects to the left ventricle by means of the mitral valve [1]. In the majority of cases it is diagnosed in neonatal period or early infancy, whereas adult cases are very rare. We report an unusual case of cor triatriatum in a 33 - year old woman presented with congestive heart failure caused by left ventricular systolic dysfunction.

## Case presentation

A 33 - year old woman presented to our cardiology service with signs and symptoms of congestive heart failure. Her medical history was unremarkable, however a year ago and soon after her third child delivery, she had been admitted in another hospital for acute pulmonary oedema after labor. Cor triatriatum with obstructive behavior causing pulmonary hypertension had bee diagnosed, while the left ventricle was structurally and functionally intact. The patient at that time denied surgey and had been discharged on medical therapy. At present admission the patient presented with NYHA functional class III, symptoms of heart failure and palpittions as a result of persistent atrial flutter. On physical examination a loud pulmonary component of the 2^nd ^heart sound and a diastolic murmur was heard in the mitral area. Signs of right-sided heart failure were absent.

A transthoracic echocardiography revealed a moderately dilated left ventricle (LV), globally hypokinetic, with severely impaired systolic function (EF estimated ≥30%). Left atrium (LA) was dilated, with a mobile, membrane-like echogenic structure into it.

**Figure 1:**
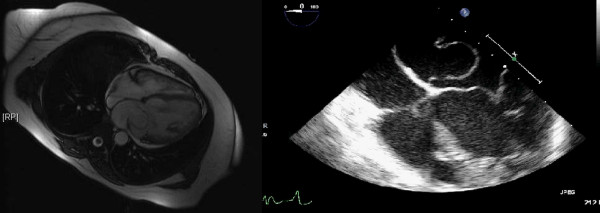
**MR of the heart cine 4-chamber view (left) showing a fibro muscular septum into the left atrium dividing it into two compartments which communicate via a central orifice (left)**. Mid-esophageal (ME) 4-chamber view (right) showing the membrane coursing transversely into the left atrium (right).

Transesophageal echocardiogram (TEE) documented a fibromuscular membrane across the LA, dividing it into two compartments, a proximal one receiving the pulmonary venous flow and a distal one containing the left atrial appendage (LAA). The two chambers communicated via a non-restrictive orifice, but the membrane prolapsed towards the mitral valve inflow causing severe obstruction. Mitral valve appeared normal, with mild regurgitation. Patent foramen ovale (PFO), atrial septal defect (ASD) and anomalous venous connections were ruled out and the diagnosis of cor triatriatum was reconfirmed. Magnetic resonance imaging ( MRI) of the heart also revealed the fibromuscular septum into the left atrium and the low left ventricular ejection fraction [(LVEF) 30%, cardiac index 1,6 L/min/m^2^, cardiac output 2,7 L/min] (figure 1). Coronary angiography showed normal coronary arteries. With these findings the patient was scheduled for surgery.

**Figure 2:**
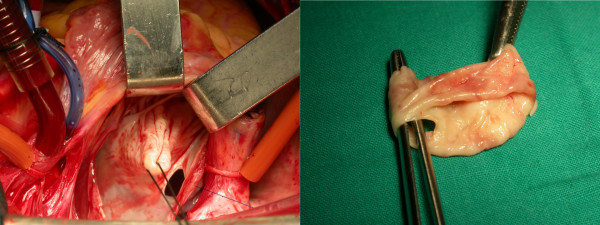
**Surgical image of the membrane in the left atrium with an eccentric opening (left)**. Completely resected membrane (right).

Anesthetic induction was achieved with standard technique including administration of sodium pentothal, sevofluorane, fentanyl and muscle relaxant. Invasive monitoring included the use of right radial arterial lines, a pulmonary artery catheter and a foley catheter with temperature probe to measure bladder temperature as an indicator of core body temperature. Transesophageal echocardiography (TEE) was also instituted. Surgery was performed through a median sternotomy. Connection to cardiopulmonary bypass (CPB) was achieved by standard ascending aorta and bicaval cannulation. Mildly hypothermic (32°C) CPB was established. Cold blood cardioplegia was administered in an antegrade fashion through the aortic root after clamping the aorta. The interatrial groove was developed and the common pulmonary venous chamber of the left atrium was opened through a vertical incision anterior to the right pulmonary veins, exactly as for mitral valve surgery. After insertion of a self-retaining retractor to facilitate exposure, the diaphragm was exposed and the central hole in it was identified. A preliminary incision out from the hole improved exposure for the definitive excision. Orifices of the pulmonary veins on both sides were located. Position of the atrial septum was also identified by a small opening in the right atrium and by inserting a curved clamp to displace the septum into the common pulmonary venous chamber of the left atrium. There was no atrial septal defect or patent foramen ovale. The diaphragm was then easily completely excised exposing the mitral valve (figure 2). The left atrial appendage was closed from inside the left atrium using a running 3-0 polypropylene suture to prevent future thrombus formation. The atriotomy incisions were closed, the heart having been filled with blood before the last few sutures were placed. The patient was rewarmed, the aortic cross-clamp was removed and additional de-airing was carried out in the usual manner. CPB was terminated with minimal inotropic support, involving milrinone and levophed with good hemodynamics.

The postoperative course was uneventful and the patient was extubated after 12 hours and discharged from the hospital on the fifth postoperative day. At 3 months follow-up, the patient was asymptomatic (NYHA class I), in sinus rhythm. TTE and MRI revealed a mildly dilated LV with great improvement in systolic function and an estimated LVEF of 50%.

## Discussion

Cor triatriatum is a rare congenital anomaly with a ratio of men to women of 1.5:1 [2]. In cor triatriatum the right and left pulmonary veins can be considered as not joining the left atrium but rather as entering a chamber posterior and a little superior or medial to the left atrium that is analogous to the common pulmonary venous sinus found in patients with total or partial anomalous pulmonary connection. However the pulmonary veins in cor triatriatum are incorporated into the structure of the left atrium, whereas in total anomalous pulmonary venous connection the pulmonary veins connect to sites separate from the left atrium. Other associated anomalies are the unrooted coronary sinus with a left superior vena cava joining the left atrium, ventricular septal defect, coarctation of the aorta, atrioventricular septal defect, tetralogy of Fallot and rarely asplenia and polysplenia. No genetic predisposition has been linked to this particular anomaly. The clinical features on presentation can mimic those of mitral stenosis, supravalvular mitral ring, left atrial thombus or pulmonary venous stenosis, since these entities share a common haemodynamic pathophysiology of flow obstruction between the pulmonary venous system and the left ventricle. The most common presenting symptoms in adults are dyspnea, hemoptysis, orthopnea as a result of the obstructive function of the intra-atrial membrane [3]. Several techniques have been used for diagnosis establishment such as TTE, TEE, CT, MRI. The use of CT bares the risk of radiation, while TEE the discomfort of scope intubation. MR imaging when compared with echocardiography and cardiac angiography was found to have a higher detection rate [4]. In addition MR fast gradient-recalled echo imaging of the cardiac cycle has been shown to be of better benefit in the assessment of cardiac function and has been established as the modality of choise for the assessment of LVEF [5]. According to Loeffler's classification of the lesion, group 3 lesions have large openings in the membrane, leading to little or no obstruction [6]. Patients with group 3 lesions can survive into adulthood with minor or no symptoms at all, as in the case of our patient. Late clinical presentations and conversion to a symptomatic state may be due to fibrosis and calcification of the orifice of the septum, onset of atrial flutter and fibrillation with rapid ventricular response, development of mitral regurgitation. Asymptomatic patients with an incidental diagnosis and a non-restrictive opening of the intra-atrial diaphragm, can be observed and followed-up regularly by TTE or MRI [7]. For symptomatic patients, surgical excision is the definite treatment, eventhough successful balloon catheter dilatation of the communication between the two chambers has been described [8].

Our patient had two previous uneventful pregnancies and experienced acute heart failure symptoms in the early postpartum period of her third normal pregnancy. The increased demands of pregnancy induce an even greater pressure gradient between the left cardiac chambers and thus a greater elevation of left atrial pressure, causing a decompensation of the patient's previously compensated cardiac function. However, severe systolic dysfunction causing symptomatic heart failure, to the best of our knowledge, has never been reported in patients with cor triatriatum.

## Conclusion

The presence of normal coronary anatomy and the exclusion of cardiomyopathies, using CMR, combined with the rapid recovery after surgical correction, leads us to believe that there is a causal relationship among these entities. Pronounced preload mismatch due to severe membrane prolapse in the LV inflow, combined with the sequential volume changes during pregnancies, leaded to decompensation and systolic dysfunction. Membrane surgical excursion leaded to rapid recovery. Peripartum cardiomyopathy seems highly unlikely, due to late onset, and rapid postoperative recovery.

## Consent

Written informed consent was obtained from the patient for publication of this case report and any accompanying images. A copy of the written consent is available for review by the Editor-in-Chief of this journal.

## Competing interests

The authors declare that they have no competing interests.

## Authors' contributions

All authors have made substantial contributions to conception and design, or acquisition of data, or analysis and interpretation of data and have been involved in drafting the manuscript or revising it critically for important intellectual content. All authors read and approved the final manuscript. JK, VA, IN: Manuscript Preparation, Study Design, Data Interpretation, Literature Search; GA, IP, NT, EP, TK: Manuscript Preparation, Literature Search, Data Acquisition; AL, AM: Manuscript Preparation, Study Design and coordination.
